# Incidence of kiaa1549-braf fusion gene in Egyptian pediatric low grade glioma

**DOI:** 10.1186/s40169-015-0052-7

**Published:** 2015-03-03

**Authors:** Hala Taha, Maha Yehia, Madeha Mahmoud, Mohamed El-Beltagy, Myret Ghabriel, Shahenda El-Naggar

**Affiliations:** Department of Pathology, Children’s Cancer Hospital Egypt 57357, P.O Box 11441, 1 Sekeat El-Imam, Cairo, Egypt; Department of Pediatric Oncology, Children’s Cancer Hospital Egypt 57357, P.O Box 11441, 1 Sekeat El-Imam, Cairo, Egypt; Department of Neurosurgery, Children’s Cancer Hospital Egypt 57357, P.O Box 11441, 1 Sekeat El-Imam, Cairo, Egypt; Basic Research Department, Children’s Cancer Hospital Egypt 57357, P.O Box 11441, 1 Sekeat El-Imam, Cairo, Egypt

**Keywords:** Glioma, *BRAF*, Cancer, Gene fusion, *KIAA1549*

## Abstract

**Background:**

Low grade gliomas are the most common brain tumor in children. Tandem duplication involving the *KIAA1549* and the *BRAF* kinase genes results in a gene fusion that has been recently characterized in a subset of low grade glioma While there is no clear evidence that the *KIAA1549*-*BRAF* gene fusion has an effect on prognosis, it is an attractive target for therapy development and as a diagnostic tool.

**Methods:**

In the current study we examine the prevalence of *KIAA1549-BRAF* gene fusion in pediatric patients diagnosed with low grade glioma in the Egyptian population and its relationship to clinical and histological subtypes. Sixty patients between the ages of 1 to 18 years were analyzed for the presence of *KIAA1549-BRAF* fusion gene products using reverse transcription-PCR and sequencing. The clinicopathologic tumor characteristics were then analyzed in relation to the different fusion genes.

**Results:**

*KIAA1549-BRAF* fusion genes were detected in 56.6% of patients. They were primarily associated with pilocytic astrocytoma (74.2%) and pilomyxoid astrocytoma (60%). Translocation 15–9 was the most common, representing (55.8%) of all positive samples followed by 16–9 (26.4%) and 16–11 (8.8%). Pilocytic astrocytomas presented primarily with 15–9 (32.2%), 16–9 (25.8%) and 16–11 (6.4%) while pilomyxoid astrocytomas presented with 15–9 (46.6%), 16–9 (6.6%) and 16–11 (6.6%) translocations.

**Conclusion:**

Gene fusion is found to be significantly increased in cerebellar pilocytic astrocytoma tumors. Furthermore, 15–9 was found to have a higher incidence among our cohort compared to previous studies. While most of the gene fusion positive pilomyxoid astrocytomas were 15–9, we find the association none significant.

## Background

Pediatric brain tumors are the second most common childhood malignancy after leukemia accounting for 25% of cases. In the last two decades the overall survival rates have improved for childhood leukemia with a 5-year overall survival of over 80%. Despite advances in surgery, chemotherapy and radiotherapy, brain tumors continue to be the leading cause of cancer-related death in children. This is mainly attributed to cellular heterogeneity of these tumors with multiple cell of origin, lack of effective drugs that cross the blood brain barrier and the absence of molecular markers that could be used for targeted therapy.

Asrtrocytoms are the most common type of brain tumors seen in children compromising 53% of tumors [1]. According to WHO criteria, which consider both pathological and clinical criteria, LGGs are classified into grade I or II. WHO grade I and II tumors are a heterogeneous group of tumors including pilocytic astrocytomas (PAs), pilomyxoid astrocytomas (PMAs), pleomorphic xanthoastrocytoma (PXA), diffuse or fibrillary astrocytoma (DA), subependymal giant cell astrocytoma (SEGA) and low grade glioneuronal tumors (LGGNs) [2]. LGG tumors are generally associated with good prognosis with a 87% over all long term survival of 20 years. Surgical excision is primary treatment method for low grade glioma within accessible parts of the brain where chemotherapy and radiotherapy are used for inoperable and or partially removed tumors [3,4]. For several years the tumor suppressor gene *NF1* which is either inherited as an autosomal disorder in patients with neurofibromatosis 1 or occur as a *de novo* mutation and tuberous sclerosis, were the only genetic factors associated with LGGs [5,6]. In recent years, several genomic alterations have been identified in sporadic low grade gliomas [7]. Deregulation of the *RAF* gene leading to constitutive activation of the MAPK pathway is emerging as a common mechanism for oncogenesis in sporadic LGG [8,9]. The activation of *RAF* has been shown to occur either through an activating point mutation (BRAF V600E), or much more frequently, through genomic alteration on 7q34 which creates a tandem duplication between the *KIAA1549* gene and the *BRAF* gene kinase domain [10,11]. As a result of this translocation the auto inhibitory domain of *BRAF* is lost and the MAPK/ERK pathway is constitutively activated in these tumors. Later studies confirmed the presence of these gene fusions, primarily in 65-75% of PAs and PMAs. Several break points were identified leading to gene fusion between *KIAA1549* exon 16 with *BRAF* exon 9 (16–9) in 60% of the cases, *KIAA1549* exon 15 with *BRAF* exon 9 (15–9) in 25% of the cases and *KIAA1549* exon 16 with *BRAF* exon 11 (16–11) in 10-15% of the cases [10]. While other fusions have been reported between exons 15–11 and 17–10, they only represented 1% of the cases. Gene fusions between *BRAF* and *FAM131B* and even less common fusions between *RAF1* and *SRGAP3* have also been reported [12]. An activating mutation at codon 600 converting valine to glutamic acid (V600E), is another mechanism for activating *BRAF* without upstream RAS phosphorylation in LGG. V600E mutation exist in diverse tumors, and have been recently identified primarily in PXA and GG and less common among PAs and PMAs tumors [13].

In the current study we investigated the prevalence of *KIAA1549-BRAF* fusion genes in a cohort of Egyptian pediatric LGG. Apart from one study which correlated *BRAF* gene fusion with poor prognosis [14], the presence of *BRAF* gene fusions have not been associated with disease outcome. However, the characterization of these fusion genes serve as a molecular biomarker for LGG subtypes and may help in selecting patients for MAPK directed therapy [15]. Our results confirm the presence of gene fusion in PAs and PMAs and to lesser extent in GG. While 16–9 gene fusion is the most common gene fusion, our results identify 15–9 *KIAA-BRAF* gene fusion as the most common in our cohort accounting for 32.2% of the positive cases where the majority of 15–9 gene fusion are seen among PMA histological subtype. Furthermore, 11% of the cases presented with both *KIAA-BRAF 16–9* and 15–9 gene fusions in the same patient.

## Methods

### Pathological specimens

Low-grade astrocytomas (WHO grades I and II) from 60 patients aged from 1 up to18 years were obtained from the Children’s Cancer Hospital Egypt (CCHE) Pathology Department. All work was conducted on excess material from the Departments of Pathology at CCHE. The research study was approved by the Institutional Review Board for Human Research at CCHE and the ethics committee waived the need for consent. All tissue samples were embedded in OCT after surgical resection and were obtained before any adjuvant chemotherapy was given. Detailed tumor subtypes and locations are summarized in Table 1. Diagnosis were made using both haematoxylin & eosin-staining and immunostaining using the following markers: GFAP, synaptophysin, EMA, MIB-1 and S-100 (Ventana, Tucson, Arizona, USA). All specimens were reviewed by two pathologists to confirm the diagnosis.

**Table 1:** ***KIAA1549*** **and** ***BRAF*** **fusion genes primer sequences**

**Primer name**	**Sequence**	**TM**
KIAA1549_15_F(A)	5′ - CGTCCTGGACAAGACCAAGT - 3′	60
KIAA1549_16_F(A)	5′ - CTCGTCTTTCTCCTGCTCGT - 3′	60
BRAF_9_R(A)	5′ - ACTCCTGACTGCATGGAAGC - 3′	60
BRAF_11_R(A)	5′ - GTCCACAGCTGCTTTTCCAC - 3′	60
KIAA1549_15_F(B)	5′ - CGTCCTGGACAAGACCAAGT - 3′	60
KIAA1549_16_F(B)	5′ - CTCGTCTTTCTCCTGCTCGT - 3′	60
BRAF_9_R(B)	5′ - ACTCCTGACTGCATGGAAGC - 3′	60
BRAF_11_R(B)	5′ - GTCCACAGCTGCTTTTCCAC - 3′	60

### RNA isolation and RT-PCR

Total RNA was isolated from 60 frozen/ fresh samples using Trizol (Invitrogen, Life Technologies, California, USA) according to manufacturer protocol. A total of six section of 50 micron were used/ sample for RNA isolation. Total RNA (500 ng to 1 μg) was used to synthesize first-strand cDNA using cDNA synthesis kit (ThermoScientific Rockford, Illinois, USA) according to the manufacturer’s instructions. One hundred nano grams of cDNA were amplified using DreamTaq Master Mix (ThermoScientific Rockford, Illinois, USA) and 10 pmole of the forward and reverse primers. Primer sets corresponding to different fusion genes were previously described and are listed in Table 1 [10,16]. Product size corresponding to different combination of primer pairs are shown in Table 2. The PCR cycling conditions were as follows: denaturation for 5 minutes at 95°C followed by 35 cycles of denaturation at 95°C for 30 seconds, annealing at 63°C for 1 minute and extension at 72°C for 1 minute, followed by final extension at 72°C for 10 minutes. Primer pairs corresponding to 15–11 translocation were initially used as screening primer to identify all possible fusion transcripts, followed by primer specific fusion transcripts using the cycling conditions below.

**Table 2:** **Different primer pairs with associated product size for different gene fusions**

**Primer pair**	**16_9**	**16_11**	**15_9**	**15_11**
15_9(A)	N	N	160	N
15_9(B)	N	N	54	N
16_9(A)	125	N	N	N
16_9(B)	64	N	N	N
15_11(A)	714	536	369	218
15_11(B)	540	362	222	44
16_11(A)	300	122	N	N
16_11(B)	232	54	N	N

### Validation of gene fusion by Sanger sequencing

PCR products were examined on 2% agarose gels to confirm the presence of a single band corresponding to the excepted size prior to sequencing. PCR products were then purified before sequencing by using ThermoScientific PCR purification kit (ThermoScientific Rockford, Illinois, USA) according to the manufacturer’s protocols. Purified products were sequenced in both directions using BigDye Terminator v3.1 Cycle Sequencing kit (Applied BioSystems, Life Technologies, California, USA)where each reaction contained 8 μl BigDye terminator mix, 5-10 ng template DNA, 3.2 μl of primer (either forward or reverse) at a final concentration of 3.2pmol, and sterile water to a final volume of 20 μl. The thermal conditions were an initial ramp to 96°C by 2.5°C per second, 96°C for 1 minute followed by 24 cycles of (96°C for 10 seconds, ramp to optimum annealing temperature for specific PCR by 1°C/second then hold annealing temperature for 5 seconds, ramp to 60°C at 1°C/second followed by 60°C for 4 minutes), ending at 12°C. Sequence reaction products were then purified by CentriSep (Princeton Separations, Inc, Nj, USA) before obtaining DNA chromatograms on a 3130 DNA Analyzer (Applied BioSystems, Life Technologies, California, USA).

### Statistical analysis

For clinical characteristics and genetic factors, analysis was performed with Fisher’s exact test (dichotomous factors). General data analysis was conducted with R programming language. All P values were based on a two-sided hypothesis, P < 0.05 was considered to have statistical significance.

## Results and discussion

**Figure 1:**
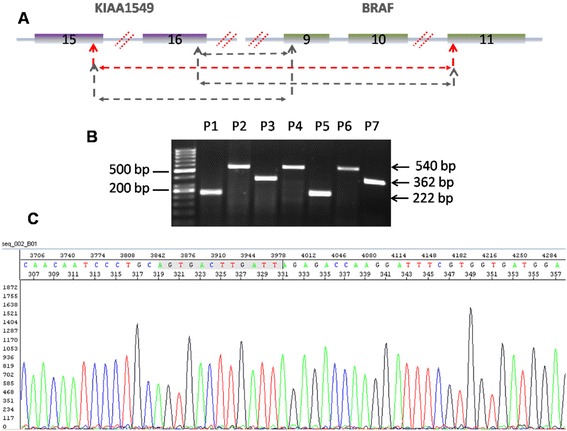
**Molecular analysis of KIAA1549-BRAF fusion genes in LGG. A**, Schematic representation of *KIAA1549-BRAF* gene fusions. Red dotted line represent common fusion sites within intron 15–16 and 16–17 of *KIAA1549* gene and 8–9, 10–11 in *BRAF* introns. Red dotted arrows represent 15–11 primer used in screening LGA samples. **B**, Identification of different gene fusions with RT-PCR using 15_F-11_R primer set showing three different product sizes corresponding to 16–9, 15–9 and 16–11 gene fusions. **C**, Partial Sequence chromatogram profile generated from fusion transcript shown in **B** confirming the fusion between 16–9, 15–9 and 16–11. Arrows indicate the junction between *KIAA1549* and *BRAF* genes.

The clinical characteristics of all 60 patients are listed in Table 3. All participating subjects were pediatric patients between the age of 1 to 18 years old with a mean of 7.7 years and median of 7 years. 83% of the cases were below the age of 12, however no association were found between patients positive for gene fusion and age range. 34 patients were males (56.6%) and 26 were females (43.3%). Patient selection was based on the availability of fresh or frozen tumor tissue for analysis. Histological examination was performed on all patients by two pathologist following the current WHO classification and resulted in 31 (51.6%) PAs, 15 (25%) PMAs, 2 (3.33%) PXA, 9 (15%) LGGN and 3 (5%) DAs. LGGN tumors included 2 (22.2%) DNT, 5 (55.5%), ganglioglioma, 1 (11.1%) desmoplastic infantile astrocytoma and 1 (11.1%) low grade glioneuronal astrocytoma, while 3 DAs tumors included low grade glioma with piloid features, diffuse glioma oligastrocytoms and diffuse fibrillary astrocytoma. PMAs have been recently identified as a separate category from PAs. These tumors tend to be more aggressive than PAs and histologically characterized by the absence of Rosenthal fibers, eosinophilic globular bodies and the presence of monomorphous piloid cells in a loose fibrillary and myxoid background [17]. A total of 37 (61.6%) tumors were cerebellar which included tumors within the vermian and the fourth ventricle, 15 (25%) were lobar, 3 (5%) were in the spinal cord, 2 (3.3%) were in the optic pathway, 2 (3.3%) were in the thalamus and 1 (1.6%) was in the brain stem. Among cerebellar tumors, 28 (75.6%) were PAs, 8 (21.6%) were PMAs and one (2.7%) was DA. The mean and median tumor size were 5.8 and 5.8 cm respectively. RT-PCR with *KIAA1549* forward and *BRAF* reverse primers located in exon 15 and exon 11 respectively were initially used to screen for different fusion genes in all samples (Figure 1B). Two different primer sets (A and B) were used to confirm the presence of gene fusions. The primer pair amplified cDNA fragments at various size corresponding to 16–9, 16–11 and 16–9 fusions (Table 2). Positive samples were then tested using a second primer set to confirm the presence of the suspected fusion as described in Table 2. Amplified products were analyzed by direct sequencing to verify the presence of the different fusions (Figure 1C). A total of 23 (74.2%, P = 0.008), 9 tumors (60%, P = 0.3) and 2 tumors (22.2%, P = 0.032) were positive for the *BRAF* fusion in PAs, PMAs and LGGN respectively. The most common fusion was 15–9 (54.2%), followed by 16–9 (34.2%) and 16-11(8.57%). Out of 31 PAs, 10 (32.2%) were positive for 15–9, 8 (25.8%) were 16–9, 3 (9.7%) were positive for double fusion 16–9 and 15–9 and 2 (6.45%) were 16–11 fusion genes while 8 (25.8%) were negative. Out of 15 PMAs, 7 (46.7%) were positive for 15–9, 1 (6.7%) for 16–9 and 1 (6.7%) for 16–11 while 6 (40%) were negative. Two LGGN tumors were positive for 15–9 where one was desmoplastic infantile astrocytoma and the second was low grade glioneuronal tumor. None of the PXAs or DAs were found to be positive for the *BRAF* gene fusion (Table 4).

**Table 3:** **Clinicopathological correlation with** ***KIAA1549-BRAF*** **fusion status in patients with LGG**

	**Total**	**Positive**	**Negative**	**p-value**
Tumor examined	60	34(56.6%)	26(43.3%)	
Tumor location				
Cerebellar	37(61%)	26(70.2%)	11(29.2%)	0.008*
Lobar	15(25%)	5(33.3%)	10(66.6%)	0.068
Spinal cord	3(5%)	2(66.6%)	1(33.3%)	1
Optic pathway	2(3.3%)	0	2(100%)	0.18
Pathology				
PAs	31(51.6%)	23(74.2%)	8(25.8%)	0.008*
PMAs	15(25%)	9(60%)	6(40%)	0.769
DAs	3(5%)	0	3(100%)	
PXA	2(3.3%)	0	2(100%)	
LGGN	9(15%)	2(22.2%)	7(77.7%)	0.032*
Gender				
Females	26(43.3%)	16(61.5%)	10(38.4%)	0.79
Males	34(56.5%)	19(55.8%)	15(44.1%)	

PAs, pilocytic astrocyoma; PMAs, pilomyxoid astrocyoma; PXA, pleomophicxantho astrocytoma; LGGN (low grade glioneuronal); DAs, (diffuse astrocytom)a. *Fisher’s exact test (P > 0.05).

**Table 4:** **Distribution of gene fusions among different LGG subtypes**

**Fusion**	**PAs**	**PMAs**	**LGGN**	**DA and PXA**	**Total**
15 - 9	10	7	2	0	19
15 - 11	0	0	0	0	0
16 - 9	8	1	0	0	9
16 - 11	2	1	0	0	3
Both	3	0	0	0	3
Total	23 of 31(74.2%)	9 of 13 (60%)	2 of 9 (22%)	0	34 of 60 (56.6%)

Fusion genes were identified as described in materials and methods. Others include Desmoplastic infantile astrocytoma, diffuse fibrillary astrocytoma, ganaglioglioma, DNT, PXA and low grade glial glioneural tumors.

**Figure 2:**
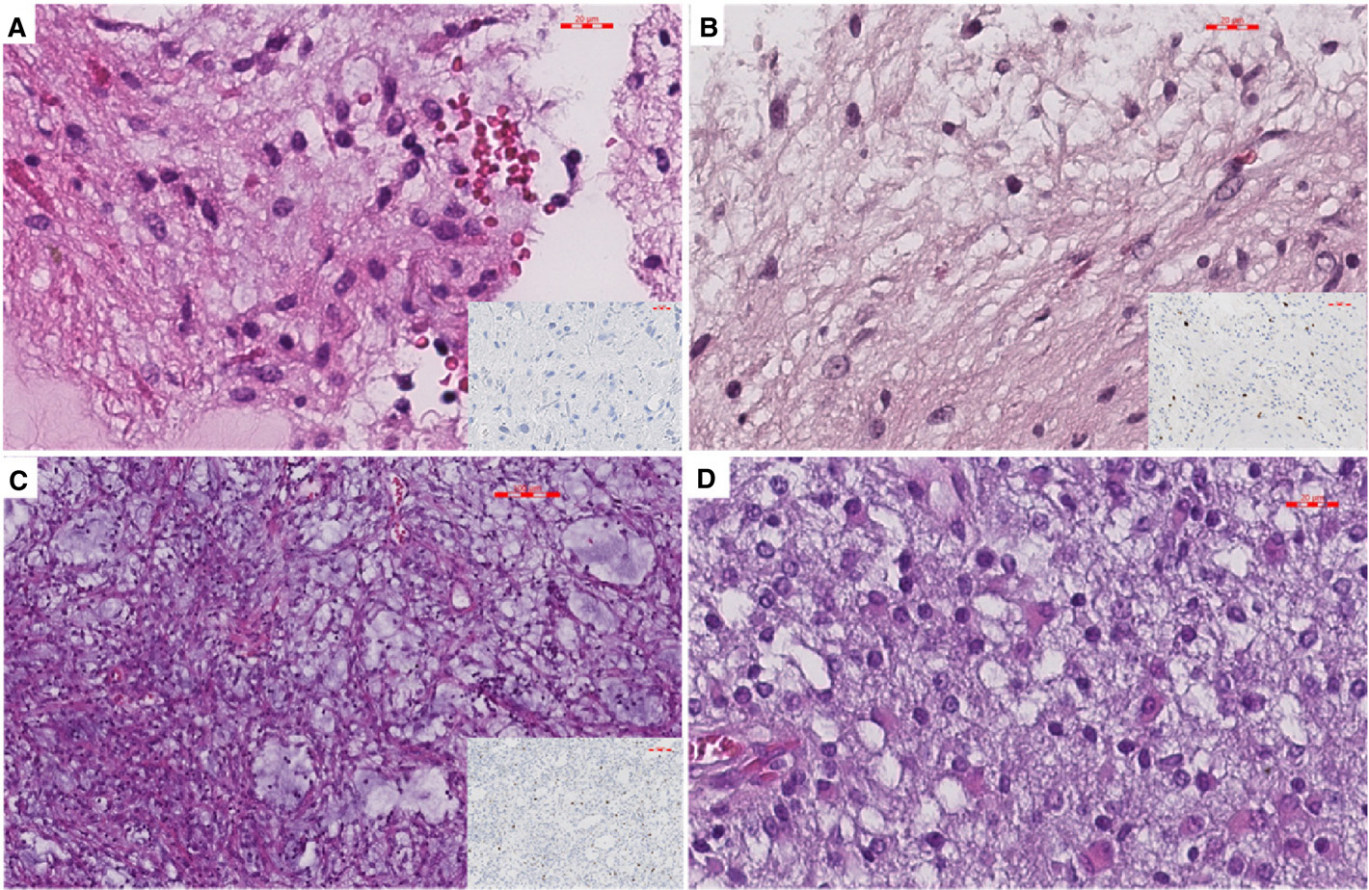
**Histological characteristics for low grade glioma with** ***KIAA1549-BRAF*** **gene fusions. A**. 16–9 gene fusion positive PAs showing a classic histology with low cellularity, minimal pleomorphism, many Rosenthal fibers, microcysts, absence of mitosis and less than 1% MIB-1 positive cells. **B**. 15–9 gene fusion PAs displaying biphasic low cellular pattern with few Rosenthal fibers and 2-3% MIB-1 index. **C**. 15–9 PMAs with myxoid background, no Rosenthal fibers, focal angiocentricity and 10% MIB-1 index. **D**. 15–9, 16–9 positive PAs with no Rosenthal fibers, myxoid background or biphasic pattern and with many gemisytocytes.

16–9 positive PAs displayed a classic histology with many Rosenthal fibers and acidophilic globular bodies (AGB) with 1-2% MIB-1 index (Figure 2A). On the other hand 15–9 positive PAs were characterized by few scattered Rosenthal fibers and AGB with occasional mitosis and higher MIB-1 index reaching 6% (Figure 2B). PMAs were characterized by the presence of myxoid background, focal to diffuse angiocentricity arrangement of cells, absence of Rosenthal fibers and high MIB-1 index reaching to 13% (Figure 2C). PAs positive for both 15–9 and 16–9 displayed a closer histology to 15–9 positive PAs (Figure 2D).

Cerebellar tumors were found to be most commonly positive for the presence of the *BRAF* fusion. Among the positive cerebellar tumors 22 (84.6%) were PAs and 4 (15.3%) were PMAs. Furthermore, 11 (42.3%) tumors were 16–9, 12 (46.15%) were 15–9 and 3 (11.5%) were 16–11. No significant difference between gender was identified with respect to *BRAF* gene fusion positive and negative patients.

Traditionally brain tumor diagnosis and classification have relied mainly on histological and clinical criteria. The use of molecular markers are growing as a diagnostic tool in brain tumors due to their potential to overcome limitations inherent to both pathological and clinical assessments [18]. The identification of the *BRAF* gene fusion highlighted the use of this genetic alteration as a potential prognostic marker and for MAPK targeted therapy [18,19]. While recent studies, investigated the effect of first generation BRAF inhibitors as a single agent both *in vitro* and phase II clinical trial were found to be not effective [20,21]. A phase I clinical trial where BRAF inhibitor Sorafenib, combined with mTOR inhibitor temsirolimus and VEGF inhibitor bevacizumab in spindle cell neoplasm showed a 25% tumor reduction in KIAA1549-BRAF positive PTEN null spindle cell neoplasm, suggesting that combined therapy may be more effective as a therapeutic alternative [22]. Several studies have investigated the significance of *BRAF* gene fusion, however despite the uncertainty for its use as a prognostic marker, the detection of *BRAF* gene fusion in low grade tumors can potentially serve as a diagnostic tool for pilocytic or pilomyxoid subtypes as it was found to be associated with these histologies in a high percentage of cases.

In the current study we aimed at identifying the incidence of *KIAA1549-BRAF* gene fusion in a cohort of pediatric Egyptian patients. Our results are in agreement with previous studies identifying *KIAA1549-BRAF* gene fusions as characteristic of both PAs and PMAs in the cerebellum. In contrast to previous reports, the15-9 gene fusion was found to be the most common finding, accounting for 55.8% of positive samples followed by 26.4% for 16–9 and 8.8% for 16–11. Furthermore, 70.2% (p value = 0.008) of cerebellar tumors were positive for the *KIAA1549-BRAF* fusion genes. The difference in distribution of the 15–9 fusion from previously reported percentages could be attributed to the population being studied [8,10]. 7 out 9 of the PMAs positive cases were positive for the 15–9 gene fusion. Although this association was found to be none significant, further investigation with larger cohort of PMAs cases is required to determine if such association exist. Three samples (8.8%) had both 15–9 and 16–9 fusion genes identified in the same tumor. These samples were tested at least twice and examined using fusion specific primer for confirmation. While duplication events and exon skipping were suggested as possible reasons for the presence of multiple junctions [16], it is not clear yet what the significance of these result are on patient prognosis nor how it may differ from single gene fusion. Histological assessment of PAs positive tumors show a difference between 16–9 and 15–9 with the former displaying a classic PA histology while 15–9 positive PAs were characterized by few Rosenthal fibers and higher MIB-1 index. Furthermore, 15–9 and 16–9 positive PAs were closer in histology to 15–9 PAs.

The use of RT-PCR for the detection for *KIAA1549-BRAF* gene fusions using two different sets of primers that spans exon 15 in the *KIAA1549* gene and exon 9 in the *BRAF* gene was found to be a reliable and rapid method that can provide confirmatory diagnosis. While FISH can provide a reliable method for the identification of *BRAF* gene fusion, it cannot distinguish between different fusion. On the other hand, RT-PCR coupled with sequencing can clearly distinguish between different fusions if needed which can be further confirmed by primer specific fusions thus allowing for more precise analysis.

## Conclusion

With the identification of new molecular markers, treatment strategies are becoming more refined. The ERK/MAPK pathway is up regulated in about a third of all cancers, in most cases this is contributed to by an activating point mutation in RAS genes [23,24]. Over the last decade, it has become apparent that activation of components of the MAPK pathway in pediatric LGG can be achieved through several distinct genetic events highlighting the importance of this pathway in LGG development. Activation of the MAPK pathway was first implicated in the development of LGG specifically in optic pathway glioma [25]. Patients with the tumor-predisposition syndrome Neurofibromatosis type 1 (NF-1) harbor a germ-line inactivating mutation in the *NF-1* tumor suppressor gene product neurofibromin which act as a negative regulator of the RAS pathway [26-28]. Inactivation of neurofibromin can also occur due to *de novo* mutation in NF-1 that have been also identified in sporadic PAs [29]. Another common way of activating the MAPK pathway is through the *BRAF* V600E mutation, which is detected in wide range of tumors such as melanomas [30], thyroid cancer [31], craniopharyngioma [32] and LGG, especially PXA and GG [13,33]. The *BRAF* V600E mutation induces a conformational change in the kinase domain which in turn results in constitutive activation of the pathway without RAS activation. Fusion genes involving *BRAF* are generated through tandem duplication and have been identified in many different LGGs, especially PAs and PMAs. In these cases, the auto inhibitory domain of *BRAF* is lost while the kinase domain is retained leading to downstream activation of the MAPK/ERK pathway [9]. These findings highlights the importance of the MAPK pathway in LGG initiation or progression and the significance of identifying patients with these genetic alterations for potential targeted therapy.

## Abbreviations

LGG: Low grade glioma
PA: Pilocytic astrocytoma
PMA: Pilomyxoid astrocytoma
PXA: Pleomophicxantho astrocytoma
AGB: Acidophilic globular bodies
DA: Diffuse astrocytoma
GG: Ganglioglioma
DNT: Dysembryoplastic neuroepithelial tumors
SEGA: Subependymal giant cell astrocytoma
LGGN: Low grade glioneuronal
NF1: Neurofibromatosis type 1
GFAP: Glial fibrillary acidic protein
EMA: Epithelial membrane antigen

## References

[1] Manuscript A, Gliomas PL. NIH Public Access. 2010;24:1397–408. doi:10.1177/0883073809342005.Pediatric.

[2] Louis DN, Ohgaki H, Wiestler OD, Cavenee WK, Burger PC, Jouvet A (2007). The 2007 WHO classification of tumours of the central nervous system. Acta Neuropathol..

[3] Sievert AJ, Fisher MJ (2009). Pediatric low-grade gliomas. J Child Neurol..

[4] Bandopadhayay P, Bergthold G, London WB, Goumnerova LC, Morales LMC, Marcus KJ (2014). Long-term outcome of 4,040 children diagnosed with pediatric low-grade gliomas: an analysis of the Surveillance Epidemiology and End Results (SEER) database. Pediatr Blood Cancer..

[5] Gutmann DH, McLellan MD, Hussain I, Wallis JW, Fulton LL, Fulton RS (2013). Somatic neurofibromatosis type 1 (NF1) inactivation characterizes NF1-associated pilocytic astrocytoma. Genome Res..

[6] Kamiryo T, Shinojima N, Ushio Y. Preliminary observations on genetic alterations in pilocytic astrocytomas associated with neurofibromatosis 1 1. 2003;228–234. doi:10.1215/S1152 10.1215/s115285170300005x PMC1920681 14565158

[7] Zhang J, Wu G, Miller CP, Tatevossian RG, Dalton JD, Tang B (2013). Whole-genome sequencing identifies genetic alterations in pediatric low-grade gliomas. Nat Genet..

[8] Pfister S, Janzarik WG, Remke M, Ernst A, Werft W, Becker N, et al. BRAF gene duplication constitutes a mechanism of MAPK pathway activation in low-grade astrocytomas. 2008;118:1739–1749. doi:10.1172/JCI33656DS1 10.1172/JCI33656 PMC2289793 18398503

[9] Forshew T, Tatevossian RG, Lawson ARJ, Ma J, Neale G, Ogunkolade BW, et al. Activation of the ERK / MAPK pathway : a signature genetic defect in posterior fossa pilocytic astrocytomas. 2009;172–181. doi:10.1002/path. 10.1002/path.2558 19373855

[10] Jones DTW, Kocialkowski S, Liu L, Pearson DM, Bäcklund LM, Ichimura K (2008). Tandem duplication producing a novel oncogenic BRAF fusion gene defines the majority of pilocytic astrocytomas. Cancer Res..

[11] Lawson ARJ, Hindley GFL, Forshew T, Tatevossian RG, Jamie GA, Kelly GP (2011). RAF gene fusion breakpoints in pediatric brain tumors are characterized by significant enrichment of sequence microhomology. Genome Res..

[12] Jones DTW, Kocialkowski S, Liu L, Pearson DM, Ichimura K, Collins VP (2009). Oncogenic RAF1 rearrangement and a novel BRAF mutation as alternatives to KIAA1549: BRAF fusion in activating the MAPK pathway in pilocytic astrocytoma. Oncogene..

[13] Dias-Santagata D, Lam Q, Vernovsky K, Vena N, Lennerz JK, Borger DR (2011). BRAF V600E mutations are common in pleomorphic xanthoastrocytoma: diagnostic and therapeutic implications. PLoS One..

[14] Hawkins C, Walker E, Mohamed N, Zhang C, Jacob K, Shirinian M (2011). BRAF-KIAA1549 fusion predicts better clinical outcome in pediatric low-grade astrocytoma. Clin Cancer Res..

[15] Ichimura K, Nishikawa R, Matsutani M. Molecular markers in pediatric neuro-oncology; 2012.90–99 10.1093/neuonc/nos204 PMC3480246 23095836

[16] Tian Y, Rich BE, Vena N, Craig JM, MacConaill LE, Rajaram V (2011). Detection of KIAA1549-BRAF fusion transcripts in formalin-fixed paraffin-embedded pediatric low-grade gliomas. J Mol Diagn..

[17] Komotar RJ, Mocco J, Carson BS, Sughrue ME, Zacharia BE, Sisti AC (2004). Pilomyxoid astrocytoma: a review. MedGenMed..

[18] Hegi ME, Murat A, Lambiv WL, Stupp R. Brain tumors: molecular biology and targeted therapies. Ann Oncol. 2006;17 Suppl:1:x191–7. doi:10.1093/annonc/mdl259. 10.1093/annonc/mdl259 17018723

[19] Manuscript A. NIH Public Access. 2012;16:103–19. doi:10.1517/14728222.2011.645805.Targeting.

[20] Karajannis MA, Legault G, Fisher MJ, Milla SS, Cohen KJ, Wisoff JH (2014). Phase II study of sorafenib in children with recurrent or progressive low-grade astrocytomas. Neuro Oncol..

[21] Sievert AJ, Lang S-S, Boucher KL, Madsen PJ, Slaunwhite E, Choudhari N (2013). Paradoxical activation and RAF inhibitor resistance of BRAF protein kinase fusions characterizing pediatric astrocytomas. Proc Natl Acad Sci U S A..

[22] Subbiah V, Westin SN, Wang K, Araujo D, Wang W-L, Miller VA (2014). Targeted therapy by combined inhibition of the RAF and mTOR kinases in malignant spindle cell neoplasm harboring the KIAA1549-BRAF fusion protein. J Hematol Oncol..

[23] Prior IA, Lewis PD, Mattos C (2012). A comprehensive survey of Ras mutations in cancer. Cancer Res..

[24] Pylayeva-Gupta Y, Grabocka E, Bar-Sagi D (2011). RAS oncogenes: weaving a tumorigenic web. Nat Rev Cancer..

[25] Rodriguez FJ, Ligon AH, Horkayne-szakaly I, Rushing J, Ligon KL, Vena N, et al. in Gliomas of the Optic Nerve Proper. 2012;71:789–794. doi:10.1097/NEN.0b013e3182656ef8.BRAF 10.1097/NEN.0b013e3182656ef8 PMC3429698 22892521

[26] Bajenaru ML, Zhu Y, Hedrick NM, Donahoe J, Parada L, Gutmann DH (2002). Astrocyte-Specific Inactivation of the Insufficient for Astrocytoma Formation Astrocyte-Specific Inactivation of the Neurofibromatosis 1 Gene ( NF1) Is Insufficient for Astrocytoma Formation.

[27] Yunoue S, Tokuo H, Fukunaga K, Feng L, Ozawa T, Nishi T (2003). Neurofibromatosis type I tumor suppressor neurofibromin regulates neuronal differentiation via its GTPase-activating protein function toward Ras. J Biol Chem..

[28] Cichowski K, Santiago S, Jardim M, Johnson BW, Jacks T (2003). . Dynamic regulation of the Ras pathway via proteolysis of the NF1 tumor suppressor. Genes Dev.

[29] Jentoft M, Giannini C, Cen L, Scheithauer BW, Hoesley B, Sarkaria J, et al. Phenotypic variations in NF1-associated low grade astrocytomas: possible role for increased mTOR activation in a subset. 2011;4:43–57 PMC3016103 21228927

[30] Hutchinson KE, Lipson D, Stephens PJ, Otto G, Lehmann BD, Lyle PL (2013). BRAF fusions define a distinct molecular subset of melanomas with potential sensitivity to MEK inhibition. Clin Cancer Res..

[31] Frasca F, Nucera C, Pellegriti G, Gangemi P, Attard M, Stella M (2008). BRAF(V600E) mutation and the biology of papillary thyroid cancer. Endocr Relat Cancer..

[32] Brastianos PK, Taylor-Weiner A, Manley PE, Johns RT, Dias-Santagata D, Thorner AR (2014). Exome sequencing identifies BRAF mutations in papillary craniopharyngiomas. Nat Genet..

[33] Schindler G, Capper D, Meyer J, Janzarik W, Omran H, Herold-Mende C (2011). Analysis of BRAF V600E mutation in 1,320 nervous system tumors reveals high mutation frequencies in pleomorphic xanthoastrocytoma, ganglioglioma and extra-cerebellar pilocytic astrocytoma. Acta Neuropathol..

